# Varus collapse following anterior closing wedge proximal tibial osteotomy for ACL revision reconstruction: a case series

**DOI:** 10.1186/s40634-022-00539-y

**Published:** 2022-10-04

**Authors:** Ian S. MacLean, William A. Tyndall, Robert C. Schenck, Mark D. Miller

**Affiliations:** 1 Department of Orthopaedic Surgery, University of Virginia Medical Center, Charlottesville, VA 22903 USA; 2 Department of Orthopaedic Surgery, University of New Mexico Health System, Albuquerque, NM 87106 USA

**Keywords:** ACL, Revision, Posterior tibial slope, Proximal tibial osteotomy, Slope correcting osteotomy, varus collapse, Malunion, Nonunion

## Abstract

A slope-correcting anterior closing wedge proximal tibial osteotomy is a powerful tool for correcting increased posterior tibial slope in the setting of a failed anterior cruciate ligament reconstruction. This case series documents three cases in which patients collapsed into varus following an anterior closing wedge proximal tibia osteotomy. Two patients had osteotomies fixated with a “suture-staple” construct, and all had medical comorbidities or reported noncompliance post-operatively. Therefore, meticulous care during the planning, execution, and rehabilitation phases is critical as multiple factors throughout the arc of care may contribute towards anterior closing wedge proximal tibial osteotomy varus collapse. Careful optimization of medical comorbidities and rigid fixation with either a plate and screws or compression staples should be used rather than a “suture-staple” to mitigate this risk.

**Level of evidence**: IV.

## Introduction

**Fig. 1:**
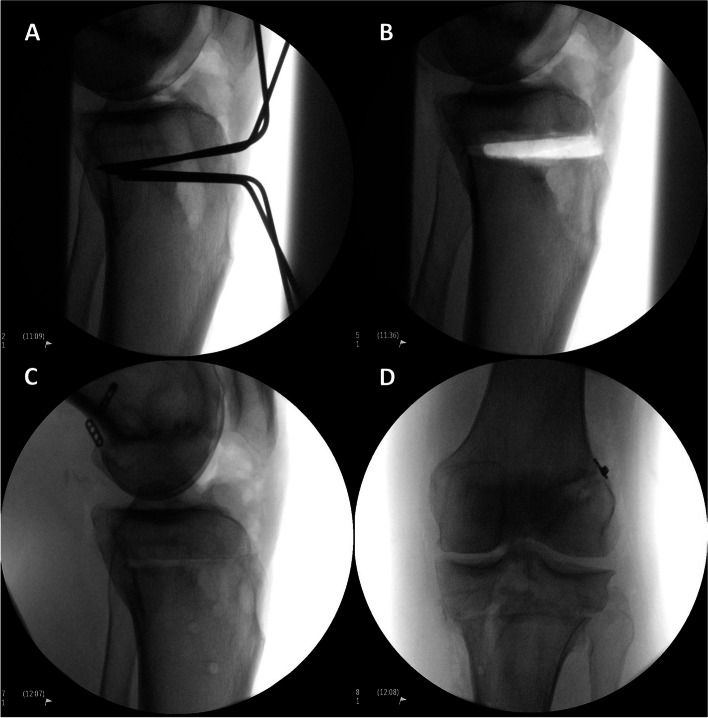
Intra-operative fluoroscopy demonstrating an anterior closing wedge PTO. Guide pins are placed (**A**). The wedge of bone is removed leaving intact the posterior cortex (**B**). The osteotomy is compressed anteriorly and fixated (**C**). Coronal alignment is unchanged (**D**)

An anterior closing wedge proximal tibia osteotomy (PTO) is an important treatment option (Fig. 1A-D) for pathologic posterior tibial slope (PTS) in the setting of anterior cruciate ligament reconstruction (ACL-R) failure. While there are many potential contributing factors for ACL-R failure, increasingly recognized is the contribution of PTS [6, 23, 27]. Numerous studies have identified a normal PTS range between 7 and 10 degrees with PTS > 12 degrees being a risk factor for graft rupture and an indication for a slope correcting osteotomy [5, 7, 17, 25, 26]. Cadaveric studies have identified increased loads on the ACL in the setting of increased PTS as well as the decreased translational forces on the ACL following an anterior closing wedge PTO [3, 7, 9, 15, 16].

The literature on clinical outcomes of proximal tibia anterior slope-correcting osteotomies is limited to several small, retrospective studies. These suggest that an anterior slope-correcting PTO may be an effective tool to minimize the risk of subsequent ACL re-rupture in appropriately selected patients [1, 5, 19]. However, the occurrence of complications is not well-reported in the literature. In this series, 10 anterior closing wedge PTOs were performed between the two institutions during the study period. We report on three cases of varus malunion collapse occurring after an anterior closing wedge osteotomy for failed ACL-R in the setting of increased PTS.

## Case reports

### Case #1

**Fig. 2:**
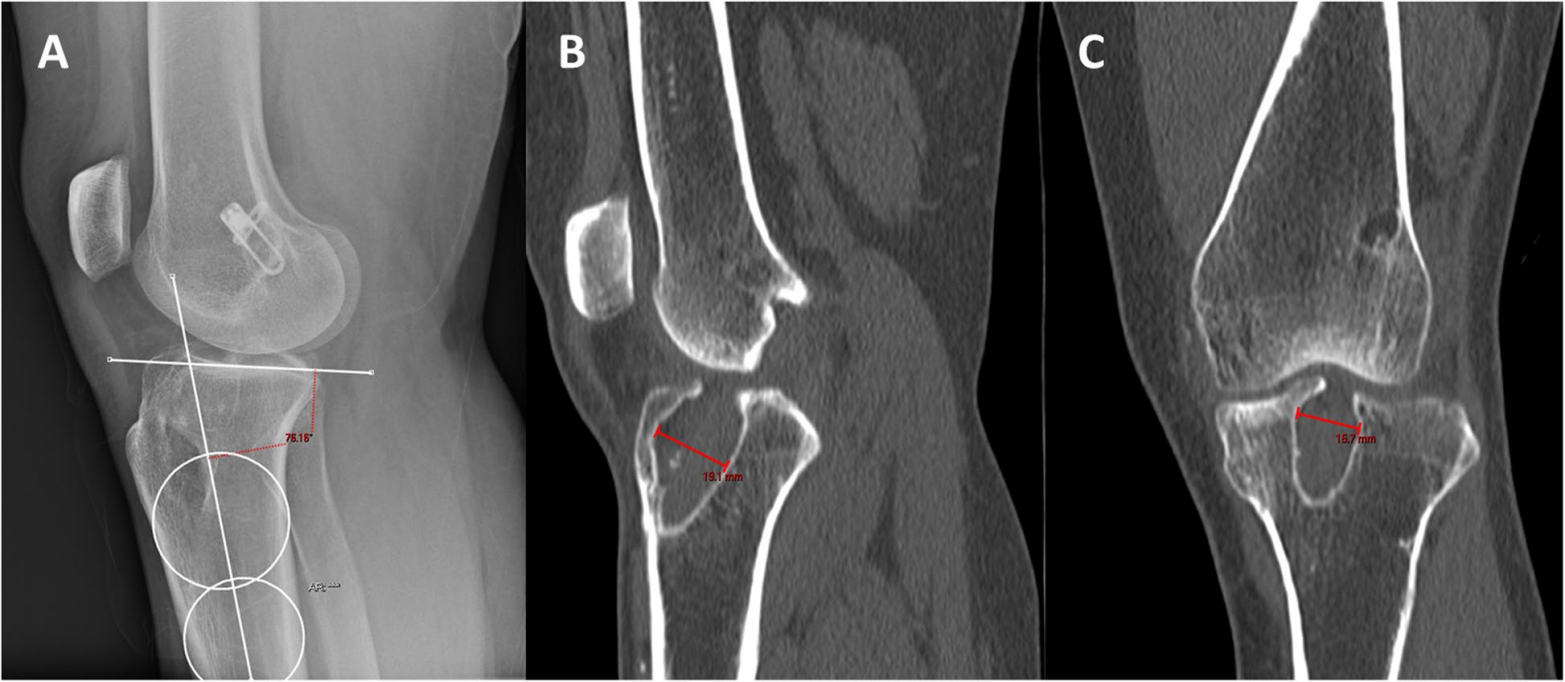
Preoperative radiograph and CT scan slices showing posterior tibial slope of 14 degrees (**A**) with tunnel widening of 19 mm on the sagittal (**B**) and 16 mm on the coronal (**C**)

**Fig. 3:**
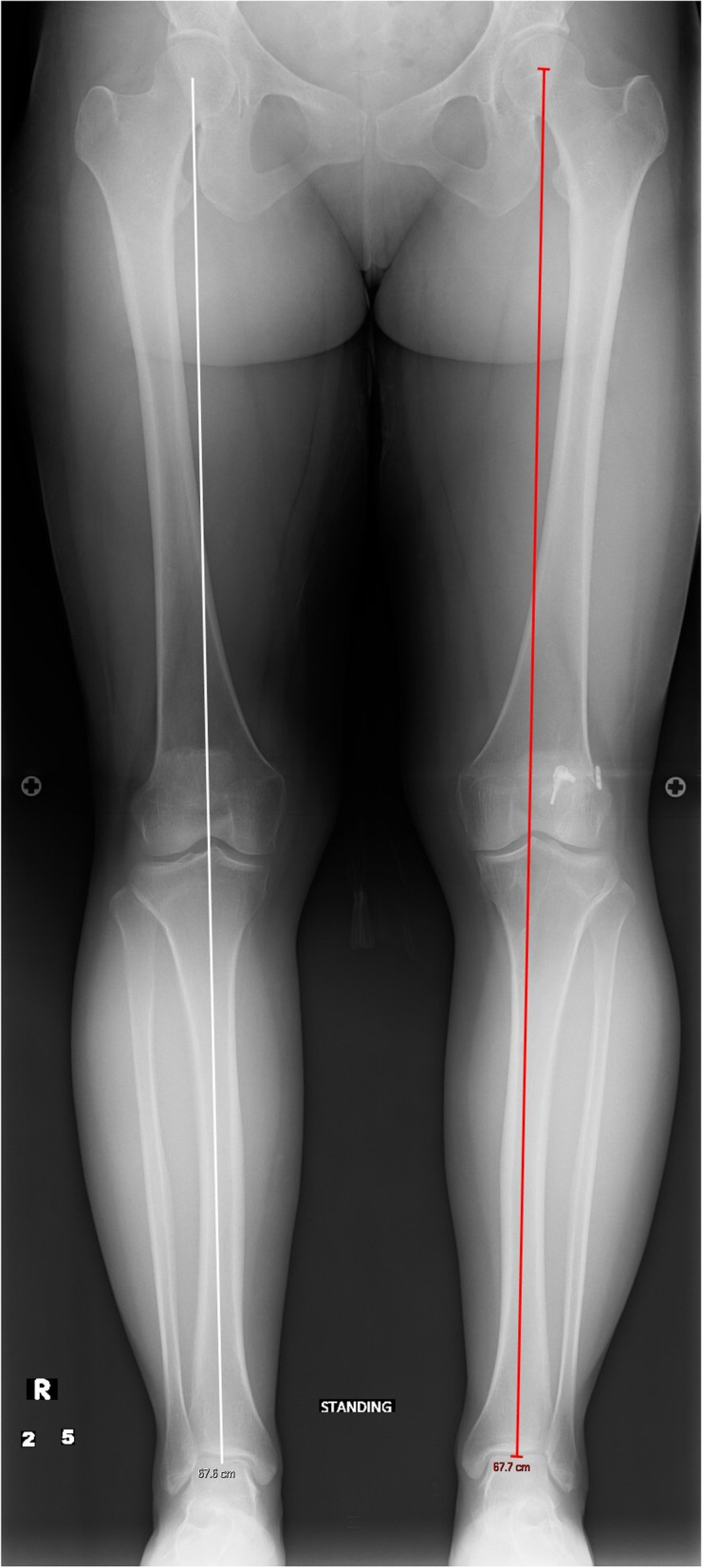
A pre-operative standing mechanical axis radiograph demonstrating physiologic varus

A 31-year-old female with a history of two prior ipsilateral ACL reconstructions presented 7 years after her previous ACL-R with about 5 years of recurrent left knee instability (Table 1). Exam and imaging were consistent with full knee range of motion (ROM), a torn ACL graft, tunnel osteolysis > 14 mm, and increased PTS (Fig. 2A-C). She had physiologic varus on mechanical axis views (Fig. 3).

**Table 1:** Patient demographics

	Case #1	Case #2	Case #3
**Age (years)**	31	39	51
**Sex**	F	M	M
**BMI**	21.6	33.6	28.5
**Smoking (PPD)**	0.5	0	0
**Comorbidities**	Depression, Anxiety, Bipolar Disorder	None	None
**Preoperative Exam**	2B Lachman; + Pivot Shift	2B Lachman; + Pivot Shift	2B Lachman
**Preoperative Tunnel Widening**	19 mm	16 mm	N/A
**Preoperative PTS**	14^o^	14^o^	17^o^
**Preoperative Mechanical Alignment**	3^o^ varus	–	3^o^ varus
**Preoperative Medial Proximal Tibial Angle**	87^o^	–	88^o^
**Previous ACL graft(s)**	1. BTB autograft 2. Hamstring allograft	1. BTB autograft	1. BTB autograft 2. Hamstring autograft
**Stage 1 Procedure**	1. Allograft bone dowel grafting of tibial & femoral tunnels 2. Anterior slope-correcting PTO	1. Arthroscopic debridement 2. Hardware removal 3. Bone dowel grafting of tibial tunnel 4. Anterior slope-correcting PTO	1. Anterior slope-correcting PTO with varus correction 2. Revision ACL-R (quadriceps tendon autograft)
**Osteotomy Fixation**	4-hole medial “X” plate; 2-hole lateral plate	Paired knotless anchors with high-strength tape-like suture on either side of the tibial tubercle	Paired knotless anchors with high-strength tape-like suture on either side of the tibial tubercle
**Stage 2 Procedure**	1. Corrective opening wedge HTO with iliac crest autograft 2. Hardware removal 3. Revision ACL-R (hamstring autograft)	1. Corrective opening wedge HTO with iliac crest autograft 2. Hardware removal 3. Revision ACL-R (quadriceps tendon autograft)	N/A

*BMI* Body Mass Index, *PPD* Packs Per Day, *PTS* Posterior Tibial Slope, *BTB* Bone-Patellar Tendon-Bone, *PTO* Proximal Tibial Osteotomy, *ACL-R* Anterior Cruciate Ligament Reconstruction, *HTO* High Tibial Osteotomy, *N/A* Not Applicable

**Fig. 4:**
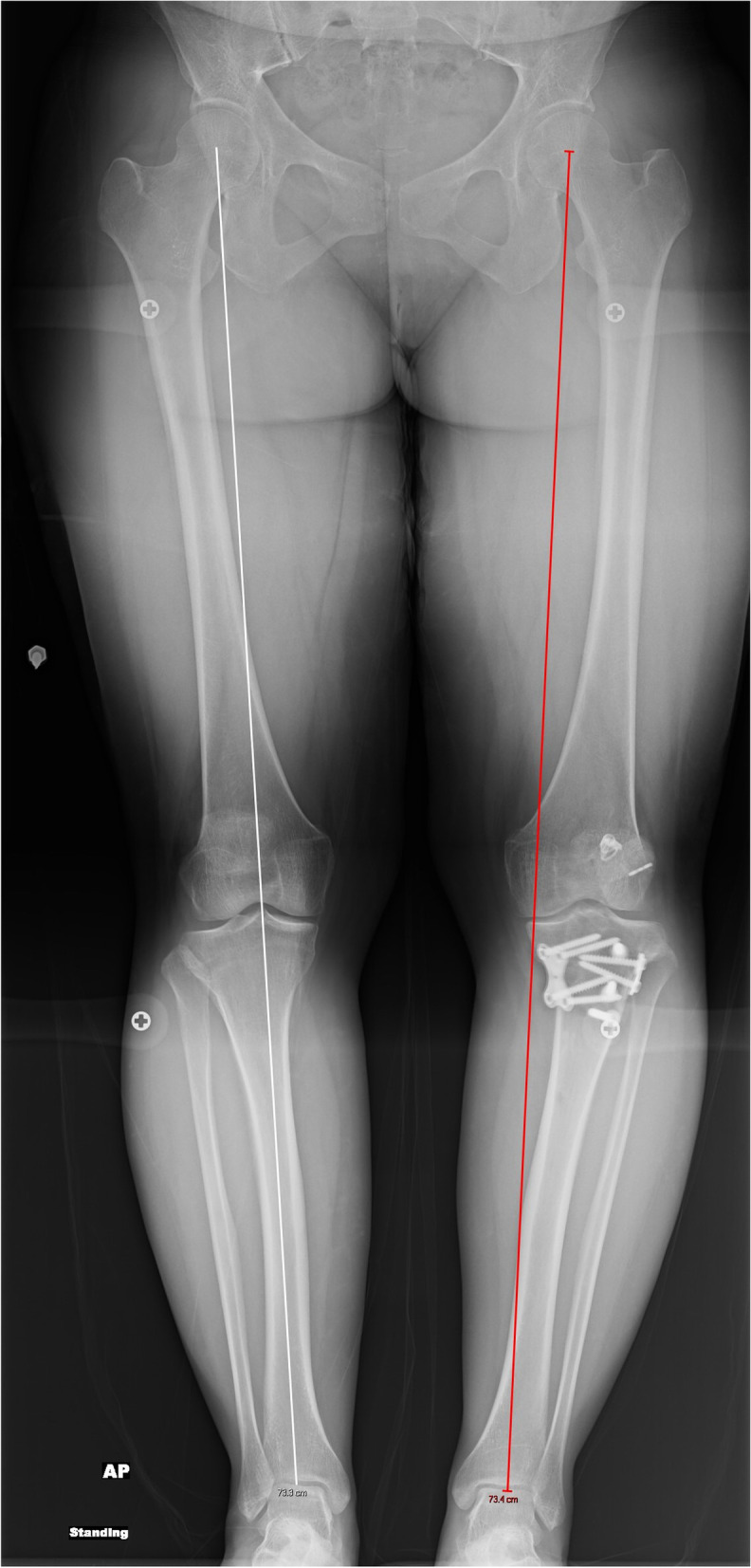
A post-operative standing mechanical axis radiograph demonstrating varus malunion of the anterior closing wedge osteotomy

She was indicated for a two-stage revision ACL-R (Table 1). There were no complications during her stage 1 procedure and the posterior cortical hinge of the osteotomy was maintained. Afterwards, patient was followed serially in clinic with interval radiographs showing correction of her posterior slope but with concern that the osteotomy was drifting into varus on mechanical axis views at 6 weeks. At 4 months, left knee films and standing mechanical alignment films were obtained which confirmed the varus malunion (Fig. 4).

**Fig. 5:**
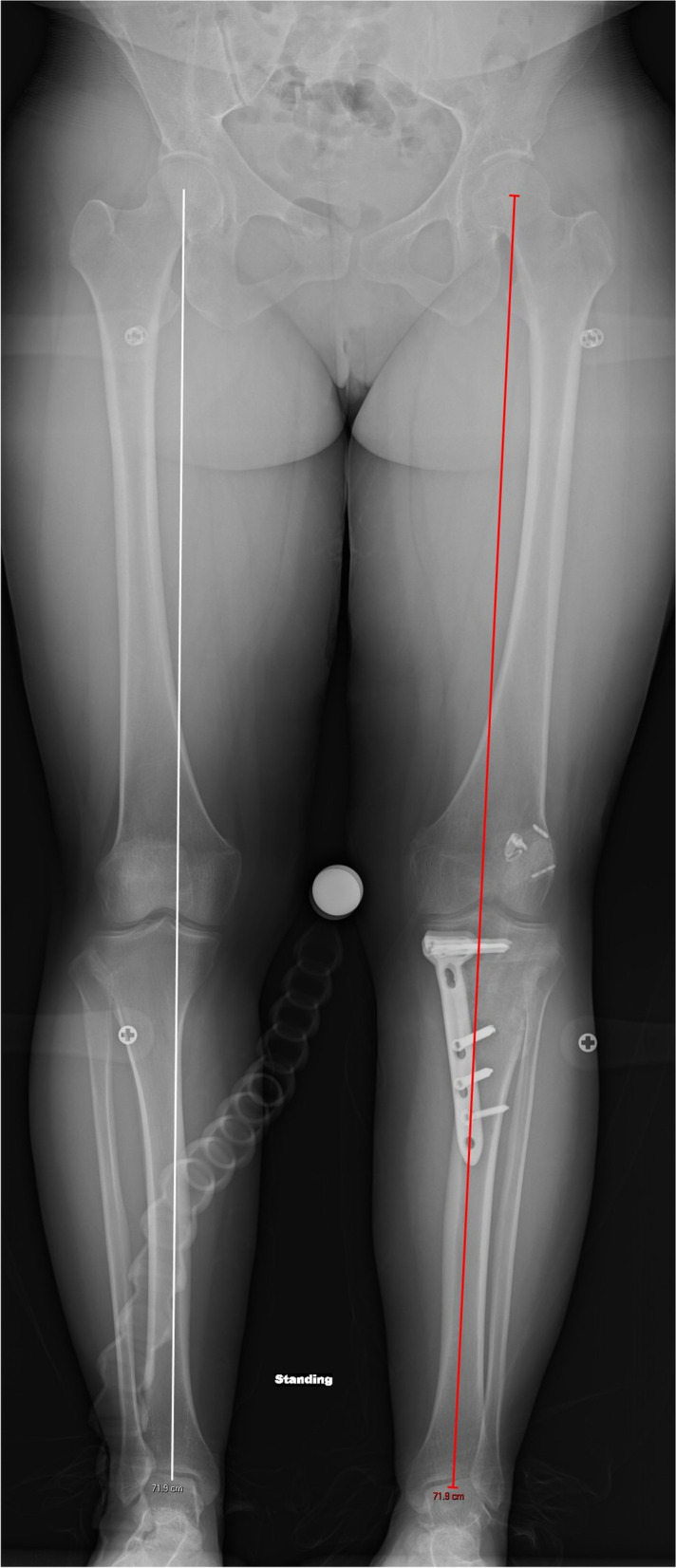
A post-operative standing mechanical axis radiograph after revision osteotomy and ACL reconstruction demonstrating neutral alignment

**Fig. 6:**
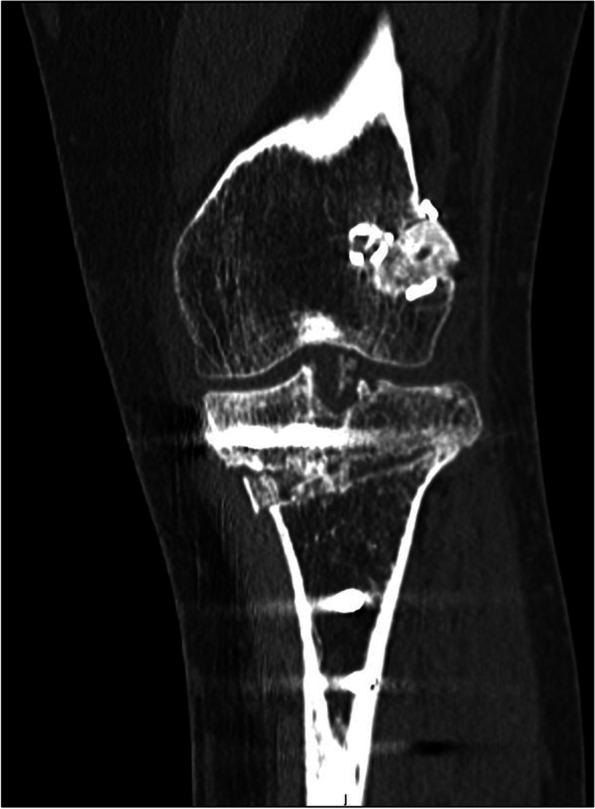
A coronal CT slice demonstrating delayed union of the osteotomy site 4 months post-operatively after the patient’s revision osteotomy and ACL reconstruction

**Fig. 7:**
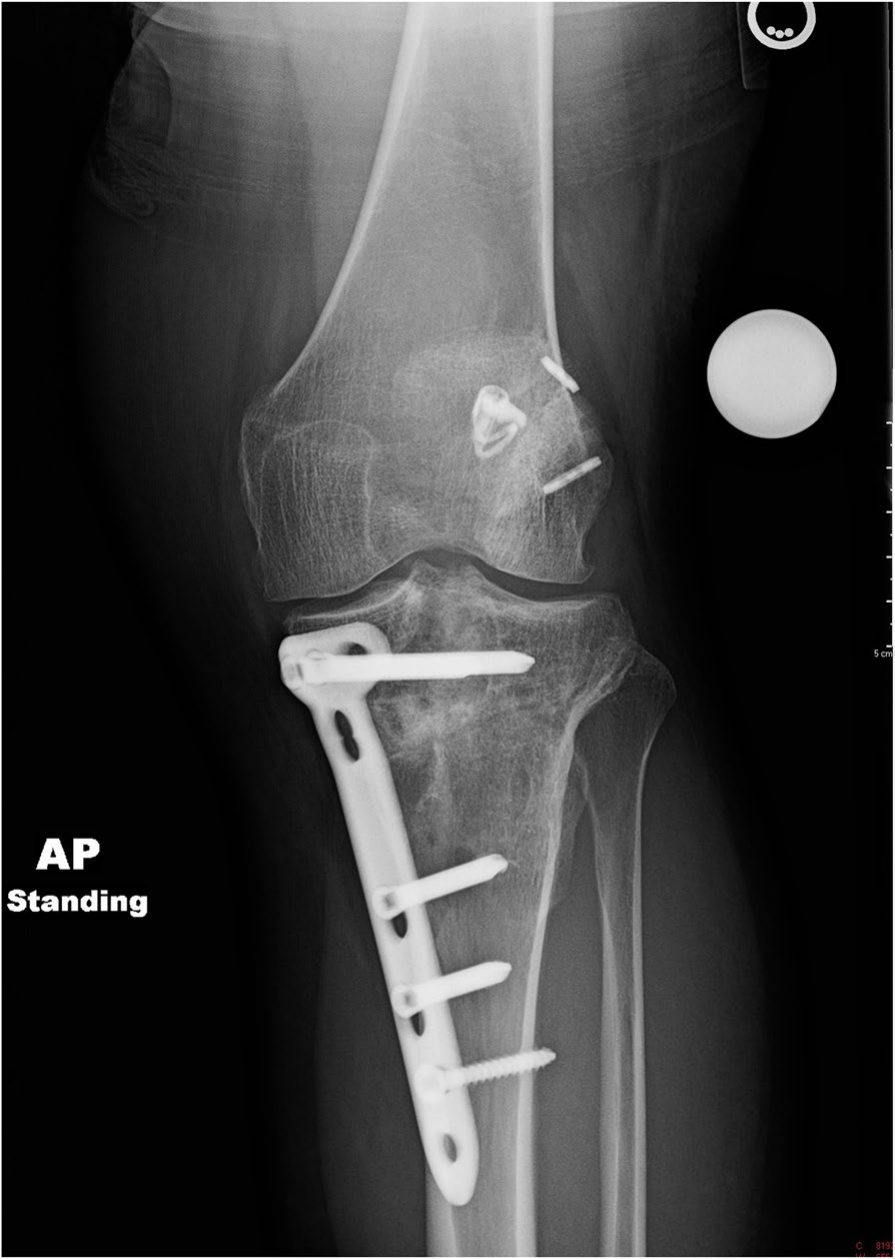
An AP radiograph showing the healed osteotomy

The patient underwent her stage 2 procedure 5 months after stage 1. This corrected her varus (Fig. 5), but a CT scan at 4 months following this showed a delayed union (Fig. 6). However, with use of a bone stimulator and close surveillance, she healed her osteotomy (Fig. 7). At final follow up, she was doing well, and her exam demonstrated no residual anterior laxity.

### Case #2

**Fig. 8:**
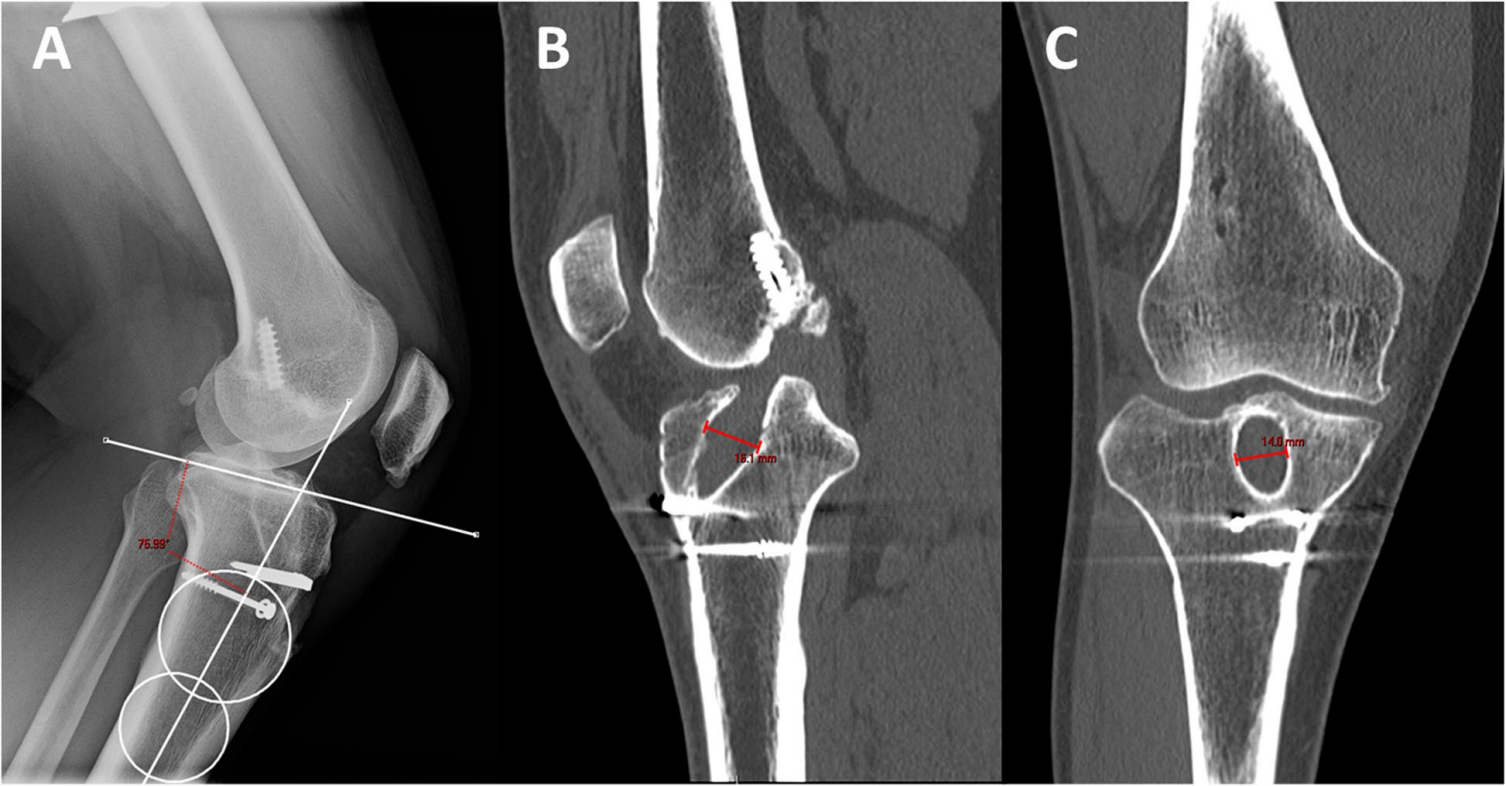
Preoperative radiograph and CT scan slices showing posterior tibial slope of 14 degrees (**A**) with tunnel widening of 16 mm on the sagittal (**B**) and 14 mm on the coronal (**C**)

A 39-year-old male with a history of one ipsilateral ACL-R 11 years prior presented with 2 months of right knee pain, swelling, and instability that occurred while boxing (Table 1). Exam and imaging were consistent with full knee ROM, a torn ACL graft, a vertical femoral tunnel, tunnel osteolysis > 14 mm, and increased PTS (Fig. 8A-C).

He was indicated for a staged ACL-R. Stage 1 occurred without intra-operative complications and the posterior cortical hinge was maintained with no gapping at the osteotomy site throughout ROM following fixation.

**Fig. 9:**
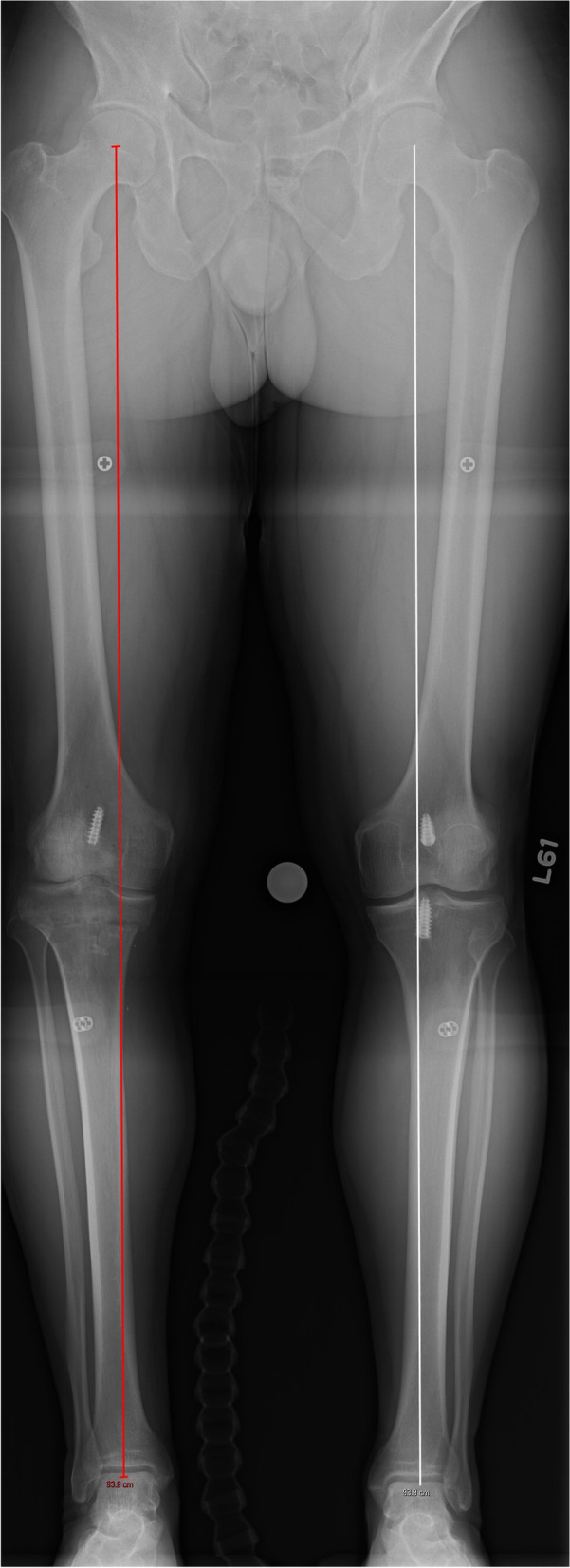
A post-operative standing mechanical axis radiograph demonstrating varus collapse of the anterior closing wedge osteotomy

**Fig. 10:**
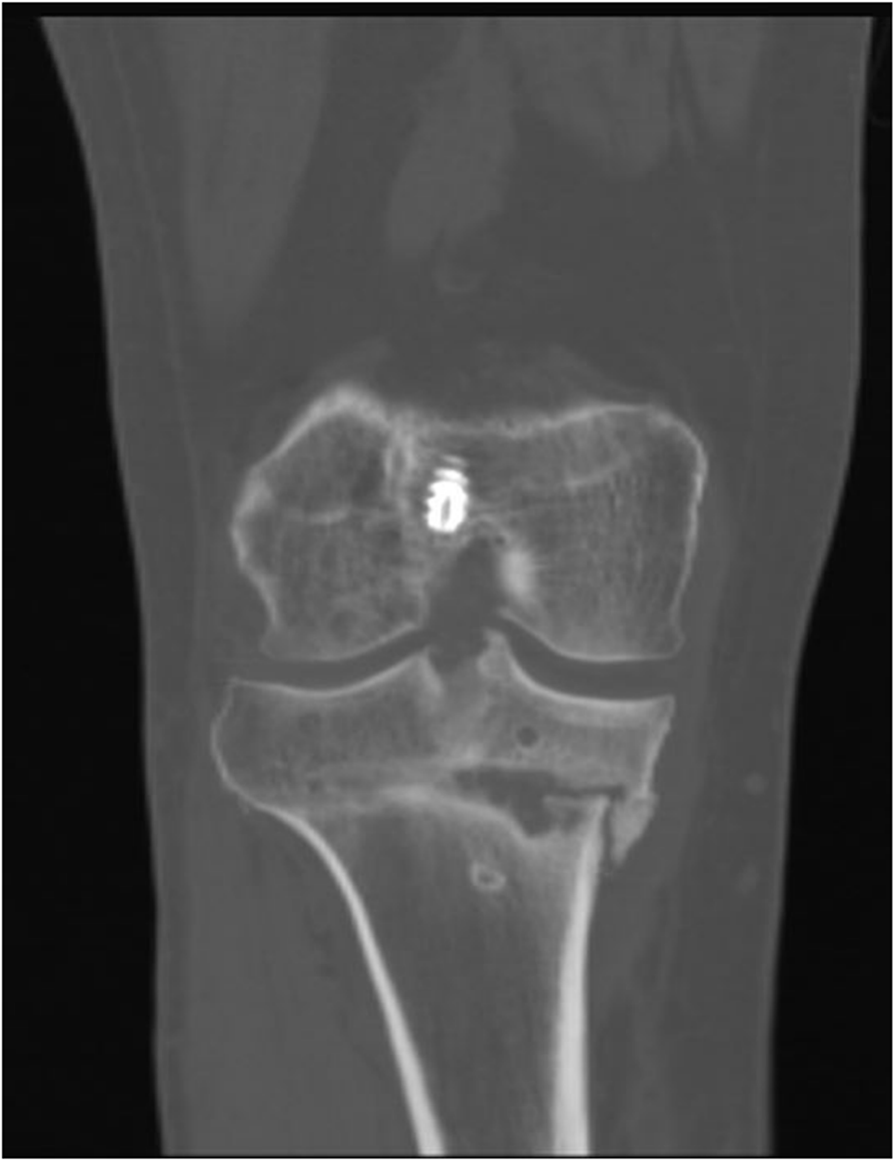
A coronal CT slice demonstrating varus collapse of the osteotomy with nonunion

A standing mechanical axis view and right knee radiographs at 6 weeks showed concern that the osteotomy had fallen into varus (Fig. 9). A 6-month CT scan showed the bone dowel allografts had incorporated. It also verified the varus collapse with areas of nonunion along the medial aspect of the osteotomy (Fig. 10). Ten months following stage 1, the patient underwent stage 2. The patient moved away a month after this and was lost to in-person follow up. However, by email he indicated he had returned to running and was doing well.

### Case #3

**Fig. 11:**
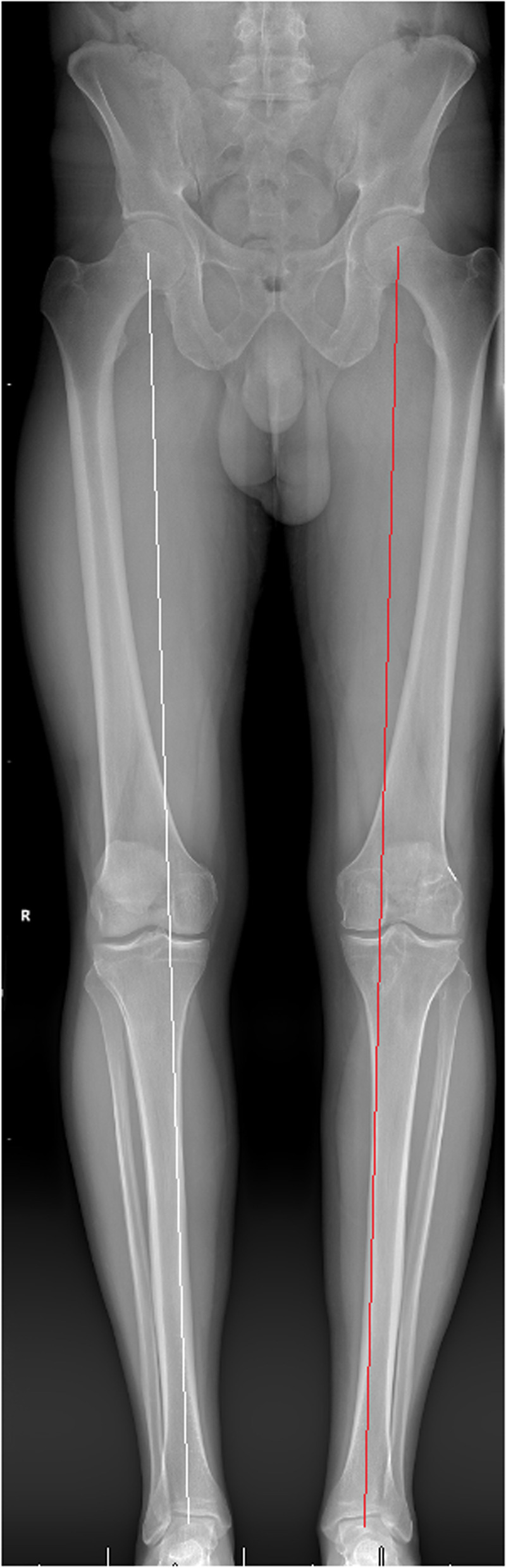
A pre-operative standing mechanical axis radiograph demonstrating slight native varus alignment

A 51-year-old male with a history of two prior ipsilateral ACL reconstructions presented 5 years after his previous ACL-R with left knee pain and instability following a pivoting injury during a baseball game. Exam and imaging confirmed full knee ROM, ACL graft rupture, and increased PTS. A preoperative mechanical axis radiograph showed slight varus alignment (Fig. 11). During the osteotomy, the posterior cortical hinge was maintained, and there was no gapping throughout ROM following fixation. A millimeter more bone was removed laterally to provide some coronal correction.

**Fig. 12:**
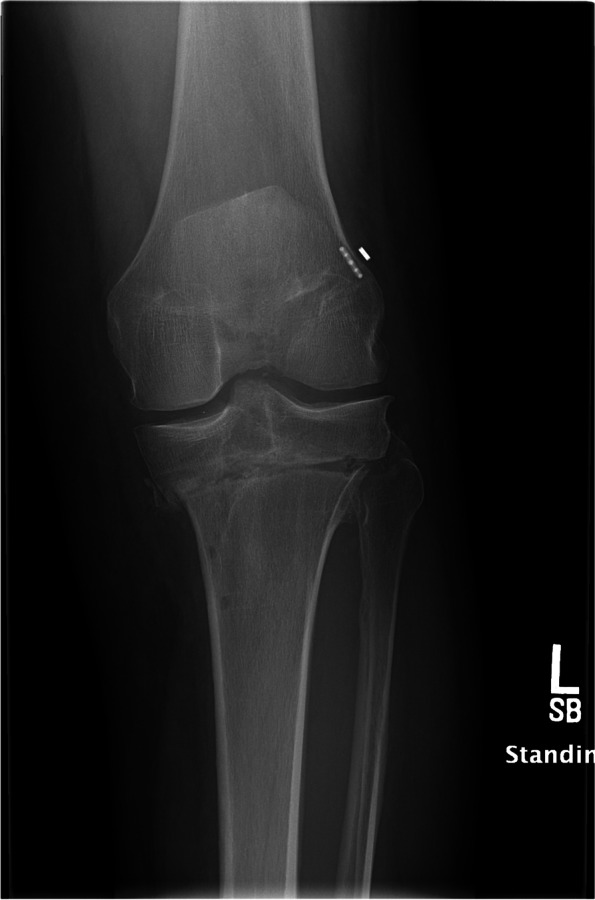
A 6-week post-operative radiograph demonstrating varus collapse of the osteotomy

**Fig. 13:**
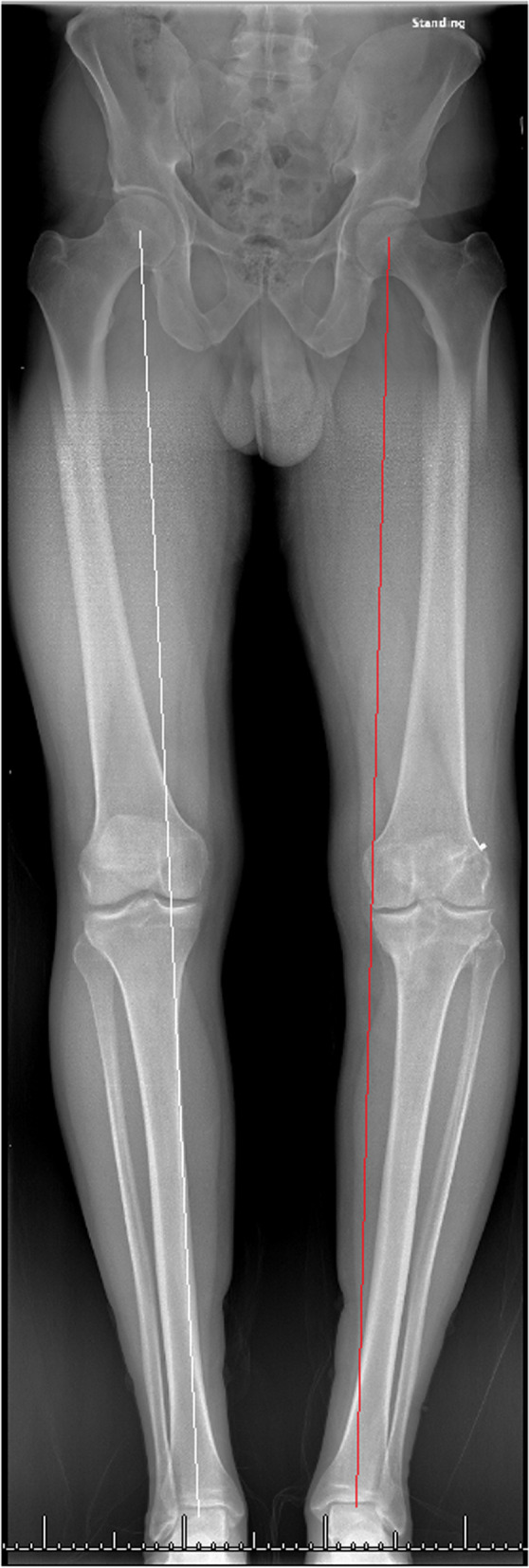
Standing mechanical axis radiograph showing a healed osteotomy without further varus progression

At the 6-week post op appointment, the patient was noted to have collapsed into varus (Fig. 12). The patient noted that he had not been able to comply with the weight bearing restrictions given his job and social factors (Table 2). The osteotomy subsequently healed without further progression (Fig. 13). The patient was able to return to basketball with a stable knee but has had difficulty returning to full running. Revision osteotomy to correct varus was discussed but the patient has not wished to proceed.

**Table 2:** Post-operative restrictions following anterior closing wedge proximal tibial osteotomy

	Weight bearing	Range of Motion	Strengthening
**Weeks 0–4**	Flat Foot (0–25%)	Weeks 0–2: 0–90 Week 2+: Progress to full	- Restore quad recruitment. - Avoid active hamstring exercises.
**Weeks 4–8**	- Weeks 4–6: 50% - Weeks 6–8: WBAT with crutches	Full	- Closed chain quad exercises. - Multi-angle knee isometrics.
**Weeks 8–16**	Full	Full	- Advance proprioceptive and balance exercises. - Progress open and closed chain strengthening. - Include elliptical and bike.

*WBAT* Weight Bearing as Tolerated

## Discussion

This case series demonstrates the importance of rigid fixation of anterior closing wedge PTOs with plate and screws or compression staples. This is especially critical in the setting of specific patient comorbidities that may elevate the risk of nonunion or malunion. A “suture-staple” construct does not provide enough stability to ensure routine healing and may risk varus collapse of the osteotomy.

Due to the infrequency with which anterior closing wedge PTOs are performed, the literature on their outcomes is limited to 4 retrospective series totaling 52 patients. A 2020 study by Akoto et al. consisting of 20 patients who underwent a 2-stage ACL revision consisting of an anterior closing wedge osteotomy in stage 1 and ACL-R with lateral extraarticular tenodesis (LET) in stage 2 noted an effective decrease in PTS, a stable ligamentous exam, and no cases of failure with an average of 30.5 months of follow up [1]. No intra-operative or post-operative complications were noted except for one patient with a post-operative hematoma which required return to the operative room on post-operative day 4. A similar series was published by Song et al. in which 18 patients with primary ACL ruptures and associated posterior horn of medial meniscus tears underwent an anterior closing wedge osteotomy for elevated PTS in the primary procedure [19]. At a minimum of 2 year follow up, all patients had stable ligamentous exams and there were no failures. No complications were noted.

A smaller series by Dejour et al. consisting of 9 patients with failed ACL-Rs also found good outcomes without failure at a mean 4-year follow up [5]. No intra-operative or post-operative complications were noted. Finally, Sonnery-Cottet et al. reported on 5 patients with combined anterior closing wedge osteotomies and revision ACL-R with a mean of 31.6 months follow up [20]. They reported no failures or complications, and all patients but one returned to pre-operative activity level. However, from the literature on concurrent high tibial osteotomy (HTO) and ACL-R, it is known there is up to a 30% complication rate and nonunion rates range from 0.7–4.4% [4, 11, 13, 21, 24].

Method of fixation varies greatly with this procedure (Table 3). If the posterior cortex is violated, a fracture plate and screw construct is necessary to ensure healing and alignment maintenance. An intact posterior hinge affords greater options. A plate with proximal locking screws provides good fixation and often greater surgeon confidence in advancing weight bearing restrictions more quickly. However, many patients find these constructs bulky and require the symptomatic hardware to be removed at a later time. Compression staple fixation offers a lower profile method that can also be used with more proximal supratubercle osteotomies with less proximal bone available for fixation. A high-strength tape-like suture and knotless anchors theoretically can be used to construct a suture-staple on either side of the tibial tubercle in much the same way a compression staple would be used. This also creates a tension band-like construct. It has been used for fixating osteotomies around the elbow and foot but has not previously been reported on for use with anterior closing wedge PTOs [2, 14, 18]. While this method avoids the issue of symptomatic hardware due to plates or staples, the authors do not recommend using this technique. Two of the 3 cases utilized this technique and failed despite being technically well-executed. Though a tension band construct with an intact posterior cortex has sufficient strength to prevent intra-operative gapping, the overall rigidity of the construct is likely insufficient to yield reliable healing especially when patients contain risk factors for nonunion.

**Table 3:** Variation in Methods for Performing and Fixating Anterior Closing Wedge Proximal Tibial Osteotomies

	Akoto et al.	Song et al.	Dejour et al	Sonnery-Cottet et al.	Current study
**Method of fixation**	“Bioplate” - screws through the TTO above and below PTO	Compression staples	Plate and screws	Compression staples for PTO & screws through TTO above and below PTO	“Suture Staple” (2); 2 & 4-hole plates with screws (1)
**Type of PTO**	Transtubercle with TTO	Supratubercle	Distal to tubercle	Transtubercle with TTO	Supratubercle

*TTO* Tibial Tubercle Osteotomy, *PTO* Proximal Tibial Osteotomy

Patient specific factors may also have contributed to the risk of varus collapse in this series. Smoking, obesity, and noncompliance with post-operative restrictions were present in cases 1, 2, and 3, respectively. Additionally, alcohol abuse, diabetes, vitamin D deficiency, and endocrine disorders all increase the risk of nonunion or malunion [8, 10, 12, 22, 28, 29]. As such, the presence of these risk factors should be screened for and any opportunity to address these and optimize their management prior to surgery should be undertaken.

In conclusion, meticulous care during the planning, execution, and rehabilitation phases is critical as multiple factors throughout the arc of care may contribute towards anterior closing wedge PTO varus collapse. Careful optimization of medical comorbidities and rigid fixation with either a plate and screws or compression staples should be used rather than a “suture-staple” to mitigate this risk.

## Abbreviations

ACL: Anterior Cruciate Ligament
ACL-R: Anterior Cruciate Ligament Reconstruction
PTS: Posterior Tibial Slope
PTO: Proximal Tibia Osteotomy
ROM: Range of Motion
